# The Relationship between Diet Quality and Acculturation of Immigrated South Asian American Adults and Their Association with Metabolic Syndrome

**DOI:** 10.1371/journal.pone.0156851

**Published:** 2016-06-14

**Authors:** Saira A. Khan, Robert T. Jackson, Bahram Momen

**Affiliations:** 1 Department of Nutrition and Food Science, The University of Maryland, College Park, Maryland, United States of America; 2 Department of Environmental Science, The University of Maryland, College Park, Maryland, United States of America; University of Rochester, UNITED STATES

## Abstract

Even though the total SA American population is increasing rapidly, there is a paucity of information on the relationship between diet quality, acculturation and health outcomes such as Metabolic Syndrome (MetS) in the low-income South Asian (SA) sub-population. Our goal was to examine diet quality, degree of acculturation and their potential influence on MetS in a diverse sample of SA Americans. A convenience sample of 401 adult SA men and women were studied using a cross-sectional study design. Volunteers from two low-income community health clinics in Maryland were interviewed by questionnaires. MetS, defined by the consensus harmonized definition by the presence of ≥ 3 of the 5 abnormal indicators, was studied. An interviewer obtained an automated self-administered 24-hour Recall (ASA24) and an acculturation index (using a previously validated (SL-ASIA). SA had a composite HEI2010 score of 68 suggesting an overall need for diet improvements. Males had a higher diet quality (mean HEI2010 score) than females. Males with MetS had lower diet quality (68) than males without MetS (73). The converse was true for females (68 vs. 65). Americanized (more acculturated) subjects had a higher diet quality compared to less acculturated SA. Small differences were found in diet quality scores among SA adults from different countries. Less acculturated females, had a higher percentage of MetS and lower diet quality compared to males. These results suggest that interventions are needed in males and females who were less acculturated because they may have greater MetS and lower diet quality compared to more Americanized SA.

## Introduction

Cardiovascular disease (CVD) is the leading cause of death in the United States of America (USA), yet little is known about its major risk indicator, metabolic syndrome (MetS), in South Asian(s) (SA) [1], who presently constitute the second fastest growing minority in the USA [2]. SA in Asia have greater premature mortality from CVD than people in Western countries and this pattern apparently continues for SA immigrants to developed countries [3]. There are numerous factors that affect CVD and metabolic disease. Many of those features have been identified in the USA population. Very few factors have been uncovered among immigrant populations such as the inter-relationships of diet quality, acculturation and their impacts on chronic diseases such as MetS.

Little information is available concerning MetS in SA. MetS is a clustering of atherogenic metabolic abnormalities that lead to CVD and T2D and is said to be present if three or more of the following five components occur in the same individual: i)abdominal obesity (waist circumference (WC) ii) elevated triglycerides, iii) low high density lipoprotein cholesterol (HDLC), iv) elevated blood pressure, and v) elevated fasting glucose [4].

The increased risk of CVD and MetS risk has been related to various lifestyle factors such as diet quality and physical inactivity [5]. Poor diet and physical inactivity are two modifiable risk factors that contribute to the epidemic of obesity and also militate towards other chronic health problems [1]. A diet high in saturated fat and excessive calories have long been associated with increased risk for CVD[6]. Diet is considered a strong, modifiable risk factor for MetS leading to CVD[7]. Research in populations from developing countries has shown increased rates of CVD and MetS that accompanies the nutrition transition (with the adoption of American dietary patterns)[6]. Yet, little is known about MetS disease among those individuals upon arrival and in the early stages in the USA. The Healthy Eating Index (HEI2010) is a measure of diet quality and conforms with the newest federal guidelines [8]. The Dietary Guidelines for Americans (DGA) are evidenced-based advice to assess the diet quality of the USA population and low-income subpopulations in dietary patterns research [9] with the goal of providing helpful advice to those at increased risk for chronic diseases. The guidelines encourage a focus on eating a healthful diet, one that focuses on foods and beverages that help achieve and maintain a healthy weight, promote health, and prevent disease [10]. HEI2010 is made up of 12 components, 9 adequacy components (total fruit, whole fruit, total vegetables, greens and beans, whole grains, dairy, total protein foods, seafood and plant proteins, fatty acids) and 3 moderation components (refined grains, sodium, empty calories). According to the Center for Nutrition Policy and Promotion (CNPP), the originators of the HEI, a score ≤ 50 indicates a poor diet while scores between 50–80 indicates a need to improve diet. Scores ≥ 80 indicate a healthy diet [11]. Studies of diet quality and chronic disease have not been conducted on a representative sample of SA Americans, since in the past, SA have not been sampled as a part of the national nutrition and health surveys. The available surveys have combined SA with East Asians (Chinese, Japanese, Koreans, etc.) and are difficult to disaggregate and therefore interpret.

To understand disease risk in SA, it is important to examine the extent to which SA have acculturated to American culture. Acculturation has been measured using the Suinn Lew Asian Identity scale (SL-ASIA) in several groups including Latinos [12–13], SA[14], East Asians [15–16]. This SL-ASIA tool measures the degree of acculturation in friendship, language and behaviors including diet. Previous acculturation studies in several groups have shown a relationship between the degree to which immigrant groups have acculturated (diet) and the presence of chronic ailments such as CVD disease [5– 6]. Studies conducted by Bharmal et. al, moreover have shown that duration of residence in the USA is associated with increased rates of hypertension [3, 17], hyperlipidemia [18], diabetes [19], and obesity among SA immigrants [3]. Still other studies suggest that duration of residence in the USA influences CVD risk [20– 21].

Obesity and MetS risk have increased in Americans [22] and also among USA immigrants due to acculturation, affluence, and urbanization [3]. There are numerous factors that affect CVD and metabolic disease. Many of those have been studied in the USA population yet very little has been done among immigrant populations such diet quality, acculturation, and their impact on MetS leading to CVD. The extent to which SA have become acculturated to the USA diet and whether this is related to MetS prevalence is unknown and there is a paucity of studies examining these relationships in low-income SA immigrants.

The objective of this study was to examine the associations between diet quality and acculturation on MetS prevalence by gender and ethnic group in a diverse sample of SA American adults.

## Materials and Methods

### Subjects

We studied MetS correlates in a convenience sample of 401 SA American adult males (n = 190) and females (n = 211) between the ages of 18–70 years. The study was conducted between June 2012 and June 2013 and represented SA from diverse countries including: Pakistan (n = 223), India (n = 71), Bangladesh (n = 67), Sri Lanka (n = 19), Iran (n = 3), Afghanistan (n = 8), and Nepal (n = 9). The subjects were unrelated SA Americans selected from two low-income community health centers in Maryland that provided free primary medical care to a predominant Muslim population. We conducted interviews (n = 401) in their native language (SK) for the 24-hour recalls and the acculturation questionnaires while socio-demographic and laboratory data were extracted from the subjects clinical files. Consecutive patient arrivals were asked to volunteer by the interviewer. Two subjects refused initial interview due to time constraints, but later agreed upon their subsequent visits. We excluded subjects with type 1 diabetes, cancer, AIDS, and women who were pregnant or breastfeeding. All patients gave informed consent before participation in the study and the research protocol was approved by the Institutional Review Board (IRB) of University of Maryland and also by the review boards of the two community centers. The participants provided written consent to participate in this study and the consent was approved by the IRB prior to the study. We have copies of the subject’s written consent and they were placed in the subjects clinical records.

### Background

Background information was gathered from clinical files including date of birth, years in the USA, reason for visit, smoking (yes/no), previous conditions diagnosed by a physician, and medication history. Physical activity was defined as activities above and beyond daily living (cleaning, cooking, and household chores), measured by four questions: 1) Do you engage in vigorous exercise? 2) How many days a week? 3) How many minutes a day? 4) What exercise do you engage in? 1 = walking, 2 = running, 3 = jogging, 4 = weight, 5 = stretch, 6 = other.

Collection and classification of MetS: The diagnosis of MetS was based on the new harmonized definition guidelines that required the presence of three or more of the following five abnormal components: i) abdominal obesity (waist circumference (WC): men ≥90 cm, women ≥80cm (Ethnic specific cut-offs for SA) (4), ii) elevated triglycerides (≥ 150 mg/dl or statins), iii) low HDL-C (<40 mg/dl in men, <50 mg/dl in women), iv) elevated blood pressure (systolic or diastolic ≥ 130/85 mmHg or use of antihypertensive medication), and v) elevated fasting glucose (>100mg/dl or hypoglycemic agents). MetS indicators were obtained from the patient clinical files. Fasting blood samples were analyzed by Lab Corp, (Burlington, NC).

WC was measured using a vinyl tape measure at the midpoint point between the iliac crest and lower rib to the nearest 0.1 cm at minimal respiration. The participant’s weight and height were measured on a Detecto promed 6129, (Thornton, CO) scale with subjects wearing light street clothing with shoes removed. Hair ornaments and buns were removed from the top of the head in order to measure stature. Weights were measured to the nearest 0.1 kg and heights to the nearest 0.1 cm. Clinical patient file with who did not meet the eligibility criteria from any of the required MetS and background variables were not included in sample.

### Collection of 24-hour dietary recalls

Diet quality and intake were assessed by using the Automated-Self-Administered-24 Hour recall (ASA24). Even though, the ASA24 can be used as a self-administered tool, we obtained this information by interviews. All interviews were performed and entered by a nutritionist who spoke the language of the participants. The methodology for assigning the foods was provided by the National Cancer Institute [23, 24]. Assigning HEI2010 scores to a set of foods required translating them into the number of food groups with the appropriate amounts of foods in each group that were consistent with the USDA Food Patterns [9] with codes provided by the USDA [25].

### Scoring and weighting of ASA24

The HEI2010 calculations have been described and published previously and the algorithms are provided on the NCI website [23– 24]. The HEI2010 components can be considered as a set of scores, each of which measures compliance to a different aspect of the DGA, and the component scores can be summed to derive a total score. The maximum number of points allocated to each component serves as a weighting factor when the component scores are summed. Most components are weighted equally at 10 points. Fruits, vegetables, and protein foods have two components (total and a subgroup) that are allotted 5 points each. Empty calories is allotted 20 points because the added sugars, solid fats, and alcohol that make up this component contribute excess calories and may displace nutrient-dense foods from the diet. HEI2010 used a density approach to set standards, per 1,000 calories or as a percentage of calories and it employs least-restrictive standards, those that are easiest to achieve among recommendations that vary by energy level, sex, and/or age. A total population score of less than 51 was considered “poor”, 51–80 “needs improvement”, and greater than 80 was “good”[11].

We calculated the HEI2010 by gender and by ethnic groups, however, due to sample size limitations, we could not carry out analyses by gender within the ethnic groups.

Assessment of Acculturation: Acculturation was measured using the previously validated Suinn-Lew Asian Self Identity Acculturation (SL-ASIA) scale. The SL-ASIA scale is a 21-item multiple-choice questionnaire used to measure the degree to which South Asians have adopted American culture. The scale was designed specifically for Asian populations yielding scores that delineate acculturation status on a scale from 1–5 [26, 27]. It covers language (4 items), identity (4 items), friendship (4 items), behaviors (5 items), generation/geographic history/enclave residence (3 items), and attitudes (1 item). A final acculturation score was calculated by dividing the total value by 21; a score ranging from 1.0–2.0 connotes low acculturation, reflecting high Asian identification, a score between 2.1–3.9 connotes biculturation and a score of 4.0–5.0, reflects high Western or American acculturation or assimilation [27]. The total score has been shown to reflect the overall level of acculturation. Due to small sample sizes in the bicultural and Western groups, we combined these groups and called them Western. Studies have shown that the final score is both reliable and valid, alpha coefficients range from 0.72–0.91(15, 26). Reliability studies show that the Cronbach’s alpha for the SL-ASIA scale for Asian Americans was between 0.91 and 0.88, reflecting high reliability [27]. A validity study showed that the SL-ASIA scores were significantly correlated with demographic information hypothesized to reflect levels of Asian American identity [27]. For example, high SL-ASIA scores were associated with having attended school in the US over a longer period of time, during which time the SL-ASIA’s Asian identity score would have been reduced. We examined the extent to which the SL-ASIA scores correlated with MetS and obesity in this sample of SAA. SL-ASIA score has been used to correlate degree of acculturation with various health risks.

### Statistical analysis

We divided the ethnic groups into four categories according to country or origin: Pakistan, India, and Bangladesh. The fourth category was called the “Pooled group”, which was formed by a combining SA from other countries (Sri Lanka, Iran, Afghanistan, and Nepal) due to a small number of subjects.

Sample size was determined by performing a power calculation based on MetS prevalence, (27%) found in previous studies of SA in the USA [28, 29]. This calculation resulted in a sample size of 400 subjects with a reasonable minimum effect of α = 0.05, a minimum power to detect that effect, and the sample size that would achieve that desired level of power was 80%.

Student t-test and analysis of variance were used to compare means of continuous variables (eg. Total component scores between “Asian” and “Western” acculturated group). Chi-square test were used to compare associations among categorized variables. Statistical analysis was performed using SAS 9.2 (SAS Institute, Cary, NC). P ≤ 0.05 was considered statistically significant. Means and standard deviations are given. Variables with small numbers, e.g., smokers (n = 6) and alcohol drinkers (n = 3) were not considered in the analysis.

## Results

Four hundred one (N = 401) SA American adults of both sexes were studied. The mean age of the sample was 48 ±11 y of age. The majority (56%) of the sample were females. There were a significantly larger percentage of Pakistani women (17%) who had not completed high school compared to Indian (5%) and Bangladeshi (7%) women. Indian and Pakistani women had significantly more abdominal obesity and higher WC means compared to Bangladeshi women (Table 1).

**Table 1:** Backhground, Acculturation, and dietary characteristics of South Asian Americans Adults (18–70 yr old) by gender (n = 361).

	Ethnicity Status					
Characteristics	Pakistani		Indian		Bangladeshi	
	Male	Female	Male	Female	Male	Female
	n = 106	n = 117	n = 34	n = 37	n = 34	n = 33
Age (y) (sd)	49(11)	48(12)	49(11)	49(12)	46(11)	49(12)
WC^a^ (cm) (sd)	98(13)	97(13)	96(11)	98(16)	93(8)	90(10)
BMI^b^ (kg/m2) (sd)	28 (5)	29 (6)	28 (5)	28 (5)	26 (4)	26 (4)
MetS^c^ (%) (n)	49 (54)	49 (58)	46 (16)	62 (24)	50 (14)	52 (14)
Education (%less than High school)	5%	17%	1%	5%	2%	7%
Acculturation Score (0–5)	1.8(0.5)	1.6(0.5)	2.0(0.5)	1.7(0.7)	1.8(0.4)	1.6(0.4)
Years in the USA(sd)	12.3 (8.6)	9.6 (8.0)	12(8.2)	12.6(10.8)	10(7.4)	7.9(8.7)
Total Calories (kcal)	1987(669)	1716(650)	1970(694)	1613(614)	2260(760)	1985(643)
Saturated Fat (kcal)	17(12)	17(9)	18(11)	14(8)	18(13)	16(10)
Protein (kcal)	84(43)	80(35)	83(38)^d^	66(33)	90(42)	92(42)
Carbohydrates (kcal)	218(79)	212(89)	242(94)	228(87)	259(99)	283(84)
Fiber (g)	20(11)	20(12)	26(13)	20(13)	26(17)	25(12)
Sodium (ug)	3626(1891)	3509(1589)	3785(1573)	3469(1588)	4344(1859)	4955(1505)
Composite HEI Score (se)	68 (1.7)		67 (2.7)		68 (2.0)	

^a^ Waist Circumference,

^b^ Body Mass Index,

^c^ Metabolic Syndrome,

^d^ Significant between gender at P<0.05.

Mean BMI, WC, HEI, acculturation scores and the percentage of MetS are displayed in Table 1 across gender and ethnic categories. MetS percentages differed as follows: Indian males (46%) and females (62%), Pakistani males (49%) and females (49%), and among Bangladeshi males (50%) and females (52%). Females had a higher percentage of MetS (54%) compared to males (48%). The percentage of MetS in Indian females exceeded that found for males and females from all three ethnic groups (Table 1).

Bangladeshi’s consumed significantly more calories than Pakistani’s (P<0.05) and Indians (Table 1), significantly more calories from carbohydrates (P<0.001), and on average more fiber (P<0.05) than Pakistani’s. Bangladeshi’s also consumed significantly more sodium (P<0.05) compared to Indian and Pakistani. Males consumed significantly (P < 0.05) more total calories, calories from carbohydrates, total fat, cholesterol, and whole grains compared to females. Indian females consumed the least calories from protein (Table 1).

The total composite HEI2010 score for SA was 68±1.2. Males had a higher score (70 ± 1.8) compared to females (66 ± 1.2) (Table 1). The HEI2010 scores for Pakistani (68 ± 1.7) and Bangladeshi (68 ± 2.0), were similar. Indian (67 ± 2.7) had a lower mean HEI score.

### Diet quality by Metabolic Syndrome and gender

We compared the diet quality of males and females with MetS and without MetS. SA males with MetS had a lower diet quality score of 68 ± 2.4 compared to those without MetS, who scored 73 ± 2.3. Component scores for dairy, seafood and plant proteins, and refined grains were lower for males with MetS (Table 2).

**Table 2:** Mean HEI-2010^a^ Component and Total Scores for Metabolic Syndrome in South Asian Americans Adults (18–70 y old) by gender (n = 401).

	Males (n = 190)				Females (n = 211)			
	MetS		No MetS		MetS		No MetS	
Component (maximum score)	Mean	SE	Mean	(SE)	Mean	(SE)	Mean	(SE)
Total Fruit (5)	4.6	0.3	4.5	0.3	5.0	0.01	4.9	0.1
Whole Fruit (5)	4.9	0.2	4.9	0.01	5.0	0.01	4.9	0.01
Total Vegetables (5)	2.4	0.3	2.8	0.4	2.0	0.3	3.2	0.4
Greens and Beans (5)	3.9	0.6	4.4	0.6	3.1	0.5	4.8	0.4
Whole Grains (10)	7.0	0.7	5.5	0.7	5.6	0.5	4.5	0.5
Dairy (10)	3.4	0.3	3.7	0.4	4.5	0.3	4.8	0.4
Total Protein Foods (5)	5.0	0.1	5.0	0.01	5.0	0.1	5.0	0.01
Seafood and Plant Proteins (5)	2.4	0.5	4.3	0.8	4.5	0.6	3.7	0.7
Fatty Acids (10)	7.3	0.8	8.5	0.9	8.9	0.6	7.0	1.6
Refined Grains (10)	0.4	0.5	2.8	0.8	.003	0.01	0.01	0.1
Sodium (10)	7.1	0.6	6.8	0.6	5.1	0.6	3.5	0.6
Empty Calories (20)	19.4	0.6	19.9	0.1	19.1	0.5	18.7	0.7
HEI2010 Total Score (100)^b^, ^c^	67.9	2.4	73.2	2.3	67.9	1.5	65.2	1.7

^a^ Healthy Eating Index-2010 Score.

^b^ Healthy Eating Index-2010 Scores: <50 poor, 50–79 needs improvement, ≥ 80 good.

^c^ The total score for total fruit is out of 5 possible points. The total points for whole grains are out of 10 points possible and the total points for empty calories are out of 20 points possible.

Conversely, females with MetS had a higher diet quality score compared to females without MetS (Table 2). Males and females with MetS had significantly lower scores for total vegetables, green and beans, and dairy. Males scored lower for seafood and plant proteins. MetS differed by age groups with older adults males (73%) and females (55%) having a higher prevalence of MetS compared to younger males (28%) and females (37%).

### Metabolic Syndrome and diet quality by acculturation

SA were divided into two acculturation groups Asian (less acculturated) and Western (Americanized) based on their mean scores on the SL-ASIA questionnaire. The less acculturated group had a higher prevalence of MetS 42% vs. 34% (Table 3) and a lower diet quality score (68 ± 1.2) compared to the Americanized SA (70± 2.2) (P<0.001) (Table 3).

**Table 3:** Metabolic Syndrome and Mean Healthy Eating Index-2010 Total Scores by Acculturation for South Asian American Adults (18–70 years old) by gender.

	Asian (Score 0–3.0)		American (Score 3.1–5)		P-Value
	Male	Female	Male	Female	
Metabolic Syndrome (%)	29%	54%	20%	48%	0.011^a^
Component (maximum score)	Score Mean (SE)		Score Mean (SE)		
HEI2010 Total Score (100)^a^	68 (1.2)		70 (2.2)		0.001^a^

^a^ p-value significant between males and females.

The less acculturated group had higher fatty acids (8.6 vs. 6.7 out of 10) component score. The Americanized group had higher dairy (4.6 vs. 3.9), seafood (4.7 vs. 3.3), and component scores. Total calories (1908 kcal ± 335 kcal), calories from carbohydrates (115 kcal ± 15 kcal) and proteins (96 kcal ± 20 kcal) were higher for the Americanized group compared to the less acculturated (1871 kcal ± 244 kcal), (112 kcal ±10 kcal), (93 kcal ± 10 kcal). Americanized females consumed more calories (1761 kcal ± 643 kcal) than less acculturated Asian females (1715 kcal ± 561 kcal). Americanized males consumed more calories (2055 kcal ± 622 kcal) than less acculturated Asian males (2027 kcal ± 536 kcal), but this difference was not significant. Americanized and less acculturated males consumed significantly more calories (P < 0.01) than females (Table 3).

## Discussion

### Metabolic Syndrome

Our study is the first to examine the percentage of MetS in a diverse sample of SA adults that included Pakistani, Bangladesh, and India. Previous studies have focused on SA Indians [29, 30]. The percentage of MetS in our SA sample was 50% and was higher in females (54%) compared to males (46%). The overall prevalence of MetS in Americans, by comparison, was reported to be 34% but was higher for African Americans (35%) and lower for Mexican Americans (33%) and non-Hispanic White Americans (33%) [31].

The percentage of MetS in our sample not only differed among the three ethnic groups, but also differed by gender within ethnic groups. Previous studies have found elevated percentages of MetS among South Asians Indians [6, 29, 32–35]. Indian females in our sample had a high percentage of MetS (62%), but this prevalence was very similar to that found in a similar study reported from India [36]. The higher percentage of MetS in these women could not be explained by their age, BMI, and education attainment which were similar across all ethnic groupings. Moreover, there were no apparent differences in calorie consumption of Indian women that would explain their greater WC and percent of MetS.

### Diet quality and Metabolic Syndrome

Our study is also the first to examine the impact of dietary quality (using HEI2010) and acculturation on the burden of MetS among low-income adults from diverse SA countries. Higher mean HEI2010 scores are thought to reflect healthier diets. The overall mean score for SA Americans was 68, while the HEI2010 score found for American adults was 53 [37], suggesting a higher diet quality in SA Americans. Previous studies examining diet have focused on single nutrients, such as proteins [38, 39], carbohydrates [40] and/or dietary fats [41]. However, single nutrients do not measure overall diet quality as well as an examination of the overall diet by HEI [42, 43].

Gender differences were apparent in diet quality between those without MetS. Males had higher diet quality scores (70) compared to females (66) and less MetS.

Pakistanis and Bangladeshis had the highest diet quality scores. The diet quality score of 67) of Indian women was slightly lower than that of Pakistani and Bangladeshi women. It is not certain if the lower diet quality or nutrient intakes were contributors to the higher prevalence of MetS in these Indian females. Wang, et al. reported that higher protein intake was associated with higher diabetes risk in SA Indians in a population based cohort of 146 SA Indians aged 45–79 years living in the San Francisco Bay Area [38]. For our study, Indian females consumed the least amount of protein compared to Indian males and females from all other ethnic groups. Bangladeshi females consumed the most calories overall compared to other ethnic groups but also had a higher diet quality score and also less MetS than Indian women.

It is not possible to discern whether the mean HEI2010 differences observed among the groups (one to three points) cause or increase risk for MetS prevalence. Moreover, we could not find studies that discuss the significance of mean HEI score differences on disease status or etiology, particularly when the mean HEI differences were only slight. Overall, our HEI scores suggest that the diet quality of SA males and females needs improvement.

The higher HEI score of SA Americans did not translate in to less MetS. That calls into question the utility of HEI as an indicator for chronic disease for this ethnic group. The increased intake of certain culturally preferred foods (such as lentils) as a part of the usual diet by SA may have inconsequentially elevated the seafood and plant protein component score to which the excessive beans and lentils are added. In addition, there were 10 points allotted for sodium. SA had a very low score for this category since a majority (96%) reported consuming daily home cooked meals rather than consuming canned and more sodium laden fast foods. Further scrutiny of the utility of the HEI2010 testing and its relationship to MetS and other chronic ailments is warranted in SA groups.

The diet quality (healthy eating index scores) did not differ significantly across age groupings (older ≥ 66years, younger 18–65 years). However, MetS did differ among age groups with the older males and females having higher than the younger adults.

The lack of culturally relevant dietary measurement tools such as that which includes cultural dishes for SA may also be a barrier to precise diet quality calculations. The most current, up to date version of the National Health Food Database that was used in this study did not include culturally specific foods or recipes, which may be needed for a more insightful dietary evaluation.

### Acculturation

Less acculturated males (29%) and females (54%) had a higher percent of MetS compared to more acculturated SA males (20%) and females (48%) respectively. Females, irrespective of acculturation status, had a higher percentage of MetS than males. The percent of MetS were very high among Indian women yet their acculturation scores were the same (Table 1). Dodani et al [19] studied the associations between acculturation (SL-ASIA) and T2D and coronary artery disease among 159 SA Indians aged 35–65 years. In contrast to our findings, Dodani et al., found that the majority of SA Indians (68%) were classified as high acculturated (Western) and had greater Coronary Artery Disease [19].

### Diet quality and acculturation

The low acculturated group had a lower diet quality and higher MetS (Table 3). The overall higher HEI2010 score of SA (higher than that of Americans) may not be indicative of lower chronic disease risk. Moreover, although the diet quality is thought to be related to chronic disease risk, there may be other unmeasured variables that impact the higher prevalence of MetS in SA Americans. For example, a large portion of our study sample did not consume foods from fast food establishments, especially meat, due to the Muslim religious requirement to eat “halal” (religiously slaughtered meats).

A strength of this study was the use of the SL-ASIA questionnaire to assess acculturation where as other studies only use years in the U.S as a proxy for acculturation. SA acculturation scores were low, <2.0 out of 5.0 for most of our study sample for all ethnic groups. Males were more acculturated than females and they had resided a greater number of years in the US. In addition, males were mostly employed outside of the home (as family providers), affording them a greater opportunity to come into contact with Americans and become more acculturated.

We expected those with greater acculturation to have lower HEI2010 scores. Garduno-Diaz studied 100 males and females (>30 years of age), and reported that dietary acculturation including altered meal patterns and may be associated with a higher prevalence of MetS in SA residing in the U.K. [44]. The Garduno-Diaz study had a small sample size with self-reported diabetes and dyslipidemia instead of direct measurements. They also used previously diagnosed T2D as a proxy for insulin resistance rather than direct measurements. They reported, a western dietary pattern with more meat was associated with a higher prevalence of MetS.

In the acculturation questionnaire, there were 2 out of 21 questions about dietary acculturation: 2) What is your food preference at home? and 2) What is your food preference in restaurants? It may be that we need to devise a dietary acculturation tool that is specific for chronic diseases that takes into account the diet of SA.

Limitations to our study were the cross sectional design that could not show causation and our sample was not representative of the entire SA population. Chronic disease causation are multifaceted and multifactorial where genes, exercise, smoking, drinking and drugs, stress and other factors play a role in the risk for the disease.

It may also be interesting to compare the results of these low-income SA with wealthy SA in order to better evaluate the role of acculturation on disease. Examining the role of CVD outcomes and mortality rates related to diet and acculturation in SA would also be interesting. Furthermore, we are still in need of representative data on SA Americans in the US to better assess the prevalence of MetS.

## Conclusion

Ours is the first study to simultaneously explore the impacts of diet quality and acculturation on the prevalence of MetS among SA Americans using previously validated instruments.

The diet quality of less acculturated SA was lower and was associated with higher percentage of MetS (42%) compared to the Americanized SA (34%). Although the diet quality score of SA “needs improvement” (i.e., < 80), they have elevated MetS percentages.

Ethnically diverse SA had similar diet quality and acculturation status but gender differences were apparent. SA males had higher diet quality scores and less MetS as opposed to females who had lower diet quality scores and higher MetS. Future research may be needed to develop a more sensitive diet quality tool to ascertain the relationship between diet and chronic disease for SA by gender.

## Supporting Information

S1 File Main Data File. (SAS)

S2 File My pyramid equivalents. (XLS)

S3 File Total food file. (XLSX)

S4 File Beans and peas macros. (SAS)

S5 File Population score macros. (SAS)
